# Effectiveness of Radiomics-Based Machine Learning Models in Differentiating Pancreatitis and Pancreatic Ductal Adenocarcinoma: Systematic Review and Meta-Analysis

**DOI:** 10.2196/72420

**Published:** 2025-07-31

**Authors:** Lechang Zhang, Dewei Li, Tong Su, Tong Xiao, Shulei Zhao

**Affiliations:** 1 Department of Gastroenterology, Shandong Provincial Hospital Affiliated to Shandong First Medical University, 324 Jingwu Weiqi Rd, Jinan, 250021, China, 86 13853121769; 2 Department of Infectious Diseases, Shandong Provincial Hospital Affiliated to Shandong First Medical University, Jinan, China

**Keywords:** artificial intelligence, differential diagnosis, imaging modalities, systematic review, meta-analysis

## Abstract

**Background:**

Pancreatic ductal adenocarcinoma (PDAC) and mass-forming pancreatitis (MFP) share similar clinical, laboratory, and imaging features, making accurate diagnosis challenging. Nevertheless, PDAC is highly malignant with a poor prognosis, whereas MFP is an inflammatory condition typically responding well to medical or interventional therapies. Some investigators have explored radiomics-based machine learning (ML) models for distinguishing PDAC from MFP. However, systematic evidence supporting the feasibility of these models is insufficient, presenting a notable challenge for clinical application.

**Objective:**

This study intended to review the diagnostic performance of radiomics-based ML models in differentiating PDAC from MFP, summarize the methodological quality of the included studies, and provide evidence-based guidance for optimizing radiomics-based ML models and advancing their clinical use.

**Methods:**

PubMed, Embase, Cochrane, and Web of Science were searched for relevant studies up to June 29, 2024. Eligible studies comprised English cohort, case-control, or cross-sectional designs that applied fully developed radiomics-based ML models—including traditional and deep radiomics—to differentiate PDAC from MFP, while also reporting their diagnostic performance. Studies without full text, limited to image segmentation, or insufficient outcome metrics were excluded. Methodological quality was appraised by means of the radiomics quality score. Since the limited applicability of QUADAS-2 in radiomics-based ML studies, the risk of bias was not formally assessed. Pooled sensitivity, specificity, area under the curve of summary receiver operating characteristics (SROC), likelihood ratios, and diagnostic odds ratio were estimated through a bivariate mixed-effects model. Results were presented with forest plots, SROC curves, and Fagan’s nomogram. Subgroup analysis was performed to appraise the diagnostic performance of radiomics-based ML models across various imaging modalities, including computed tomography (CT), magnetic resonance imaging, positron emission tomography-CT, and endoscopic ultrasound.

**Results:**

This meta-analysis included 24 studies with 14,406 cases, including 7635 PDAC cases. All studies adopted a case-control design, with 5 conducted across multiple centers. Most studies used CT as the primary imaging modality. The radiomics quality score scores ranged from 5 points (14%) to 17 points (47%), with an average score of 9 (25%). The radiomics-based ML models demonstrated high diagnostic performance. Based on the independent validation sets, the pooled sensitivity, specificity, area under the curve of SROC, positive likelihood ratio, negative likelihood ratio, and diagnostic odds ratio were 0.92 (95% CI 0.91‐0.94), 0.90 (95% CI 0.85‐0.94), 0.94 (95% CI 0.74‐0.99), 9.3 (95% CI 6.0‐14.2), 0.08 (95% CI 0.07‐0.11), and 110 (95% CI 62‐194), respectively.

**Conclusions:**

Radiomics-based ML models demonstrate high diagnostic accuracy in differentiating PDAC from MFP, underscoring their potential as noninvasive tools for clinical decision-making. Nonetheless, the overall methodological quality was moderate due to limitations in external validation, standardized protocols, and reproducibility. These findings support the promise of radiomics in clinical diagnostics while highlighting the need for more rigorous, multicenter research to enhance model generalizability and clinical applicability.

## Introduction

Pancreatic ductal adenocarcinoma (PDAC) is the most common histological subtype of pancreatic cancer and represents the deadliest solid malignancy, with a 5-year survival rate below 10%. It currently ranks as the third leading cause of cancer-related mortality worldwide, posing a substantial public health burden [1,2]. Surgical resection remains the only potentially curative treatment option. However, due to its insidious onset and lack of specific early clinical signs, most patients are diagnosed at an advanced stage when curative surgery is no longer feasible [3]. Notably, early-stage or incidentally detected PDAC is associated with markedly improved survival outcomes [4]. These observations highlight the urgent need for effective diagnostic strategies for PDAC [5].

Mass-forming pancreatitis (MFP), a focal pancreatic lesion that may arise from chronic pancreatitis or manifest as autoimmune pancreatitis, poses a diagnostic challenge, especially when it mimics the clinical features of PDAC [6,7]. The 2 conditions often share overlapping clinical manifestations, laboratory profiles, and imaging characteristics, thus complicating accurate differentiation in routine practice [8,9]. However, their prognoses and treatment approaches differ significantly [10]. PDAC is characterized by aggressive biological behavior, including neural invasion and hematogenous spread, often resulting in a poor prognosis. In contrast, MFP is a benign inflammatory condition, with approximately 80% of cases responding well to medical or interventional therapy. Misdiagnosis may therefore lead to either unnecessary surgery for benign MFP or delayed intervention for malignant PDAC, both carrying significant clinical consequences.

Traditionally, endoscopic ultrasound-guided fine-needle aspiration (EUS-FNA) has served as a cornerstone for diagnosing pancreatic lesions by providing cytopathologic confirmation. Although generally safe and effective [11,12], EUS-FNA may yield inconclusive results if the samples are insufficient, particularly in the absence of rapid on-site evaluation [13,14]. In addition, tumor heterogeneity and sampling error can contribute to false-negative results [15-17]. As an invasive procedure, EUS-FNA also carries inherent procedural risks and limitations. Consequently, there is growing interest in developing noninvasive, accurate diagnostic alternatives.

Radiomics has emerged as a promising noninvasive approach that extracts high-throughput quantitative imaging features, often imperceptible to the human eye, from standard-of-care radiological scans [18]. These high-dimensional data can be processed by machine learning (ML) algorithms to develop predictive models for disease classification and characterization. Compared with conventional imaging interpretation, which is often subjective and experience-dependent, radiomics offers an objective, reproducible method for capturing intratumoral heterogeneity [19]. Its capacity to capture subtle imaging patterns also positions it as a potential complement or alternative to invasive diagnostic techniques such as EUS-FNA. Recent studies have explored the application of radiomics-based ML models for differentiating PDAC from MFP. Despite the growing number of individual investigations, systematic evidence supporting the diagnostic performance of radiomics-based ML models for detecting PDAC remains lacking. This knowledge gap presents a challenge for the development of intelligent diagnostic tools. Therefore, this study aims to systematically review the diagnostic accuracy of radiomics-based ML models in distinguishing PDAC from MFP. By synthesizing current evidence, our findings may clarify the potential clinical utility of radiomics and provide an evidence-based foundation for the development of intelligent diagnostic technologies in pancreatic diseases.

## Methods

### Overview

This study was executed and documented in alignment with the guidelines set by the PRISMA (Preferred Reporting Items for Systematic Reviews and Meta-Analyses) (Checklist 1) [20]. Furthermore, this study followed an a priori–created protocol registered on the International Prospective Register of Systematic Reviews (CRD42024575745).

### Eligibility Criteria

The eligibility criteria are described in Textbox 1.

Textbox 1. **Inclusion and exclusion criteria.**

**Inclusion criteria:**

- Studies involving patients diagnosed with pancreatic ductal adenocarcinoma or mass-forming pancreatitis, including chronic pancreatitis and a focal type of autoimmune pancreatitis.
- Studies that fully developed and applied radiomics-based machine learning models—whether based on handcrafted features (traditional radiomics) or deep learning feature extraction (deep radiomics)—for differentiating pancreatic ductal adenocarcinoma from mass-forming pancreatitis.
- Studies published in English.
- Studies with cohort, case-control, and cross-sectional designs.

**Exclusion criteria:**

- Conference abstracts not published in full.
- Studies that only performed image segmentation.
- Studies without any outcome measures to appraise the prediction accuracy of models, including c-index, sensitivity, specificity, accuracy, precision, confusion matrix, *F*_1_-score, or calibration curves.

### Data Sources and Search Strategy

A comprehensive retrieval was carried out across PubMed, Embase, Cochrane, and Web of Science from their inception to June 29, 2024. The search strategy incorporated both Medical Subject Headings terms and keywords (Multimedia Appendix 1) to ensure a thorough identification of relevant studies.

### Study Selection and Data Extraction

The collected study reports were added to an EndNote library (EndNote 20.6 [Clarivate]), where duplicates were identified and removed. To recognize relevant studies, 2 reviewers (LZ and TS) independently filtered out the titles and abstracts. The entire texts of these articles were then reviewed to assess eligibility according to the pre-established criteria. A high level of agreement was achieved between the 2 reviewers (Cohen κ coefficient=0.89). Discrepancies, if any, were resolved through discussion or adjudication by a third reviewer (SZ).

The extracted data encompassed the title, first author’s name, publication year, country, study design, patient source, source of radiomics, imaging protocol, number of imaging investigators, repeat measurement information, region of interest segmentation software, total number of PDAC cases, total number of cases, training set details (including number of PDAC cases and total number of cases), validation set details (including validation strategy, number of PDAC cases, and total number of cases), variable selection methods, model types, radiomics score construction, overfitting assessment, availability of code and data, and model evaluation metrics.

Two reviewers (LZ and TS) independently extracted the data and cross-checked the results for consistency. Any discrepancies were addressed through discussion. A third reviewer (SZ) would make the final decision if no consensus was reached.

### Assessment of Study Quality

Using the radiomics quality score (RQS), 2 investigators (DL and TX) independently appraised the methodological quality and risk of bias of the included studies [21]. The RQS assessed 16 dimensions across 6 domains, namely image and segmentation, feature selection, validation and utility, model performance, high-level evidence, and open science and data, to appraise the methodological rigor of the construction of radiomics-based ML models. The total score, varying from −8 to 36, was derived from these 16 dimensions. Upon completion, 2 investigators (DL and TX) cross-checked their evaluations. A high level of agreement was observed between them (intraclass correlation coefficient=0.97; 95% CI 0.93‐0.99). Any discrepancies were resolved through discussion or consultation with a third investigator (SZ).

### Synthesis Methods

The meta-analysis was conducted for diagnostic performance metrics, including sensitivity, specificity, area under the curve of summary receiver operating characteristic (SROC AUC), positive likelihood ratio (PLR), negative likelihood ratio (NLR), and diagnostic odds ratio (DOR), using a bivariate mixed-effects model. This statistical approach considers both within-study variability (random effects) and between-study heterogeneity (fixed effects). It is particularly suitable for meta-analyses on the accuracy of diagnostic tests, as it allows for the simultaneous estimation of sensitivity and specificity, while addressing the inherent link between multiple measures. Unlike a hierarchical SROC model, a bivariate model directly yields pooled estimates of sensitivity and specificity with CIs, better aligning with our goal of producing clinically interpretable summary measures. By employing this model, reliable estimates of diagnostic performance metrics were obtained, providing a robust basis for evaluating radiomics-based ML models [22].

During this meta-analysis, sensitivity and specificity were analyzed using the diagnostic fourfold table. Nevertheless, the diagnostic fourfold table was not provided in most of the original studies. In such cases, we derived it from sensitivity, specificity, precision, and case numbers. Although only a minority of studies explicitly reported the probability threshold used for classification (like 0.5 or thresholds based on the Youden index), most provided diagnostic performance metrics. This suggested that some form of thresholding is applied, even if it was described in detail. To address this interstudy variability in threshold selection, we used a bivariate mixed-effects model, which inherently accounts for such differences and ensures the robustness of the pooled estimates.

The included studies used ML classifiers and reported results from various sets, including training, independent validation, and cross-validation sets. Therefore, these sets were analyzed separately. To further examine the predictive performance of radiomics-based ML models, subgroup analyses were executed based on various imaging modalities, including computed tomography (CT), endoscopic ultrasound (EUS), positron emission tomography-computed tomography (PET-CT), and magnetic resonance imaging (MRI). Each subgroup was examined separately using a bivariate mixed-effects model to obtain pooled sensitivity and specificity estimates. Heterogeneity among the included studies was appraised through the Cochrane Q-test (*P* value≤.05) and *I*^2^ statistic (>50%). Publication bias was examined using a funnel plot. 95% CIs were provided for all estimates. The meta-analysis was executed using Stata 16 software (Stata Corporation).

## Results

### Literature Search

**Figure 1.**
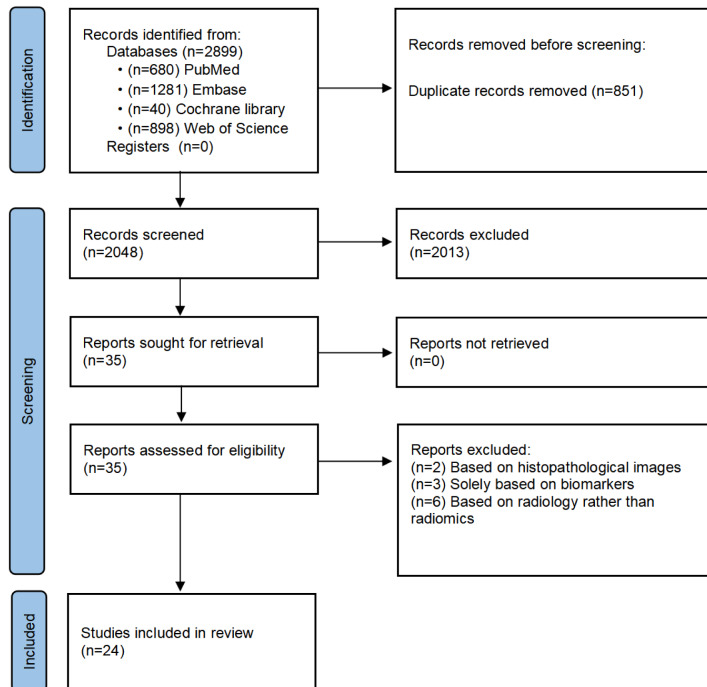
Literature screening process.

Initially, 2899 articles were identified. Of the total, 851 articles were deleted due to duplicates. From the 2048 remaining articles, 2013 were removed after screening the titles and abstracts. Upon reviewing the entire text, 11 articles were removed (2 studies based on histopathological images, 3 focusing solely on biomarkers, and 6 relying on radiology rather than radiomics). Ultimately, 24 studies were included in this systematic review [23-46]. The literature screening process is presented in Figure 1.

### Study Characteristics

The 24 included studies were published between 2019 and 2024. In total, 14,406 participants were reported, with 7635 diagnosed as PDAC (Tables1  2). All studies used a case-control design, with 5 conducted across multiple centers [26,31,35,40,42] and the remaining at single centers. CT was the most frequently used imaging modality, featured in 13 studies [23,25,26,28-32,34,36-38,42]. EUS was used in 5 studies [40,43-46], MRI in 3 studies [27,35,41], and PET-CT in 3 studies [24,33,39].

**Table 1.** Study characteristics.

Author	Year	Country	Study design	Study setting	Imaging modality	ROI^a^ segmenters, n	ROI segmentation software
Ren et al [23]	2020	China	Case-control	Single-center	CT^b^	—^c^	ITK-SNAP
Zhang et al [24]	2019	China	Case-control	Single-center	PET^d^-CT	6	3D Slicer
Zhang et al [25]	2022	China	Case-control	Single-center	CT	2	3D Slicer
Ye et al [26]	2023	China	Case-control	Multicenter	CT	2	3D Slicer
Shiraishi et al [27]	2022	Japan	Case-control	Single-center	MRI^e^	2	syngo
Ren et al [28]	2019	China	Case-control	Single-center	CT	3	ITK-SNAP
Qu et al [29]	2023	China	Case-control	Single-center	CT	2	3D Slicer
Park et al [30]	2020	United States	Case-control	Single-center	CT	7	Velocity AI
Ma et al [31]	2022	China	Case-control	Multicenter	CT	2	MITK
Lu et al [32]	2023	China	Case-control	Single-center	CT	2	LIFEx
Liu et al [33]	2021	China	Case-control	Single-center	PET-CT	2	3D Slicer
Li et al [34]	2022	China	Case-control	Single-center	CT	2	3D Slicer
Deng et al [35]	2021	China	Case-control	Multicenter	MRI	2	IBEX
E et al [36]	2020	China	Case-control	Single-center	CT	2	Weasis
Anai et al [37]	2022	Japan	Case-control	Single-center	CT	4	LIFEx
Ziegelmayer et al [38]	2020	Germany	Case-control	Single-center	CT	2	ITK-SNAP
Wei et al [39]	2023	China	Case-control	Single-center	PET-CT	3	3D Slicer
Tong et al [40]	2022	China	Case-control	Multicenter	EUS^f^	5	labelme
Chen et al [41]	2024	China	Case-control	Single-center	MRI	2	3D Slicer
Cao et al [42]	2023	China	Case-control	Multicenter	CT	48	—
Udriştoiu et al [43]	2021	Romania	Case-control	Single-center	EUS	—	—
Nakamura et al [44]	2024	Japan	Case-control	Single-center	EUS	7	—
Marya et al [45]	2021	United States	Case-control	Single-center	EUS	7	—
Kuwahara et al [46]	2023	Japan	Case-control	Single-center	EUS	—	—

a ROI: region of interest.

b CT: computedtomography.

c Not available.

d PET: positron emission tomography.

e MRI: magnetic resonance imaging.

f EUS: endoscopic ultrasound.

**Table 2.** Model details.

Author	Number of PDAC^i^ cases	Number of cases	Number of PDAC cases in the training set	Number of cases in the training set	Validation strategy	Number of PDAC cases in the validation set	Number of cases in the validation set	Model type	Model calibration quality
Ren et al [23]	79	109	79	109	Cross-validation	79	109	RF^b^	—^c^
Zhang et al [24]	66	111	66	111	Cross-validation	66	111	RF, Adaboost^d^, and SVM^e^	—
Zhang et al [25]	71	138	59	103	Prospective external validation	12	35	LR^f^	Decision curve
Ye et al [26]	120	198	84	139	Prospective external validation	36	59	LR	Calibration curve and decision curve
Shiraishi et al [27]	77	105	77	105	Cross-validation	77	105	SVM	—
Ren et al [28]	79	109	79	109	Random sampling	30	40	LR	—
Qu et al [29]	201	255	175	213	Random sampling	26	42	LR	Decision curve
Park et al [30]	93	182	60	120	Random sampling	33	62	RF	—
Ma et al [31]	151	175	151	175	Cross-validation	151	175	LR	Calibration curve and decision curve
Lu et al [32]	64	96	45	67	Random sampling	19	29	LR, RF, SVM, and DT^g^	Calibration curve and decision curve
Liu et al [33]	64	112	64	112	Cross-validation	64	112	SVM	—
Li et al [34]	42	97	42	97	Cross-validation	42	97	LR	—
Deng et al [35]	96	119	51	64	Institutional external validation	45	55	SVM	—
E et al [36]	51	96	51	96	Cross-validation	51	96	RF	—
Anai et al [37]	30	50	30	50	Cross-validation	30	50	SVM	—
Ziegelmayer et al [38]	42	86	42	86	Cross-validation	42	86	RF	—
Wei et al [39]	64	112	64	112	Cross-validation	64	112	DL^h^	—
Tong et al [40]	414	558	264	351	External validation	150	207	DL	—
Chen et al [41]	62	93	55	82	Random sampling	7	11	DL	—
Cao et al [42]	4706	9939	1431	3208	Random sampling and external validation	3275	6731	DL	—
Udriștoiu et al [43]	30	65	30	65	Cross-validation	30	65	DL	—
Nakamura et al [44]	61	85	61	85	Cross-validation	61	85	DL	—
Marya et al [45]	292	583	170	336	Random sampling	122	247	DL	—
Kuwahara et al [46]	680	933	518	694	Random sampling	162	239	DL	—

a PDAC: pancreatic ductal adenocarcinoma.

b RF: random forest.

c Not available.

d Adaboost: adaptive boosting.

e SVM: support vector machine.

f LR: logistic regression.

g DT: decision tree.

h DL: deep learning.

Regarding the software used for region of interest segmentation, the majority of studies [24-26,29,33,34,39,41] used 3D Slicer (Brigham and Women’s Hospital). ITK-SNAP (University of Pennsylvania) was used in 3 studies [23,28,38], while 2 studies [32,37] used LIFEx (Institut Curie). Other software tools mentioned included syngo (Siemens Healthineers) [27], Velocity AI (Varian Medical Systems) [30], MITK (German Cancer Research Center) [31], IBEX (MD Anderson Cancer Center) [35], Weasis (open source) [36], and labelme [MIT CSAIL] [40]. Most studies reported the methods used for variable selection, with least absolute shrinkage and selection operator regression and minimum redundancy maximum relevance being the most commonly employed techniques. In addition, the most frequently applied ML for constructing radiomics-based models was logistic regression [25,26,28,29,31,32,34], decision tree [32], support vector machine (SVM) [24,32,33,35,37], adaptive boosting [24], and random forest (RF) [23,24,30,32,36,38]. Some studies also used deep learning (DL) [39-46] models. Only 4 studies [28,31,33,35] constructed models combining clinical features with radiomics features.

All studies described their validation methods. A total of 19 studies used internal validation [23,24,27-34,36-39,41,43-46], of which 7 used random sampling [28-30,32,41,45,46], while 12 used internal cross-validation [23,24,27,31,33,34,36-39,43,44]. Furthermore, 4 studies applied prospective or external institutional validation [25,26,35,40], and 1 incorporated both random sampling for internal validation and external institutional validation [42]. The training set from all included studies comprised a total of 6689 cases, with 3748 being PDAC cases. The validation set, on the other hand, contained 8960 cases, of which 4674 were PDAC cases.

### Assessment of Study Quality

The RQS was applied to assess the 24 studies, varying from 5 points (14%) to 17 points (47%), with an average score of 9 (25%) (Multimedia Appendix 2).

The primary factors contributing to the lower quality included no phantom models across scanners to evaluate interscanner variability, no imaging data at multiple time points to assess temporal stability, and a limited correlation between radiomic features and biology. In addition, few studies reported on model calibration or conducted cost-effectiveness analyses. Most studies were retrospective in nature, with code and data generally not accessible to the public, limiting reproducibility.

Conversely, the included studies demonstrated strengths in other areas. Many studies provided well-documented imaging protocols, and a substantial number of studies used multiple segmentation methods to enhance the robustness of segmentation. Statistical techniques, such as feature reduction to avoid overfitting and discrimination statistics, including SROC curves and AUC, were widely used across these studies. Furthermore, several studies validated their models using independent datasets, which supported the clinical applicability of their radiomics-based models.

### Meta-Analysis

#### Training Set

**Figure 2.**
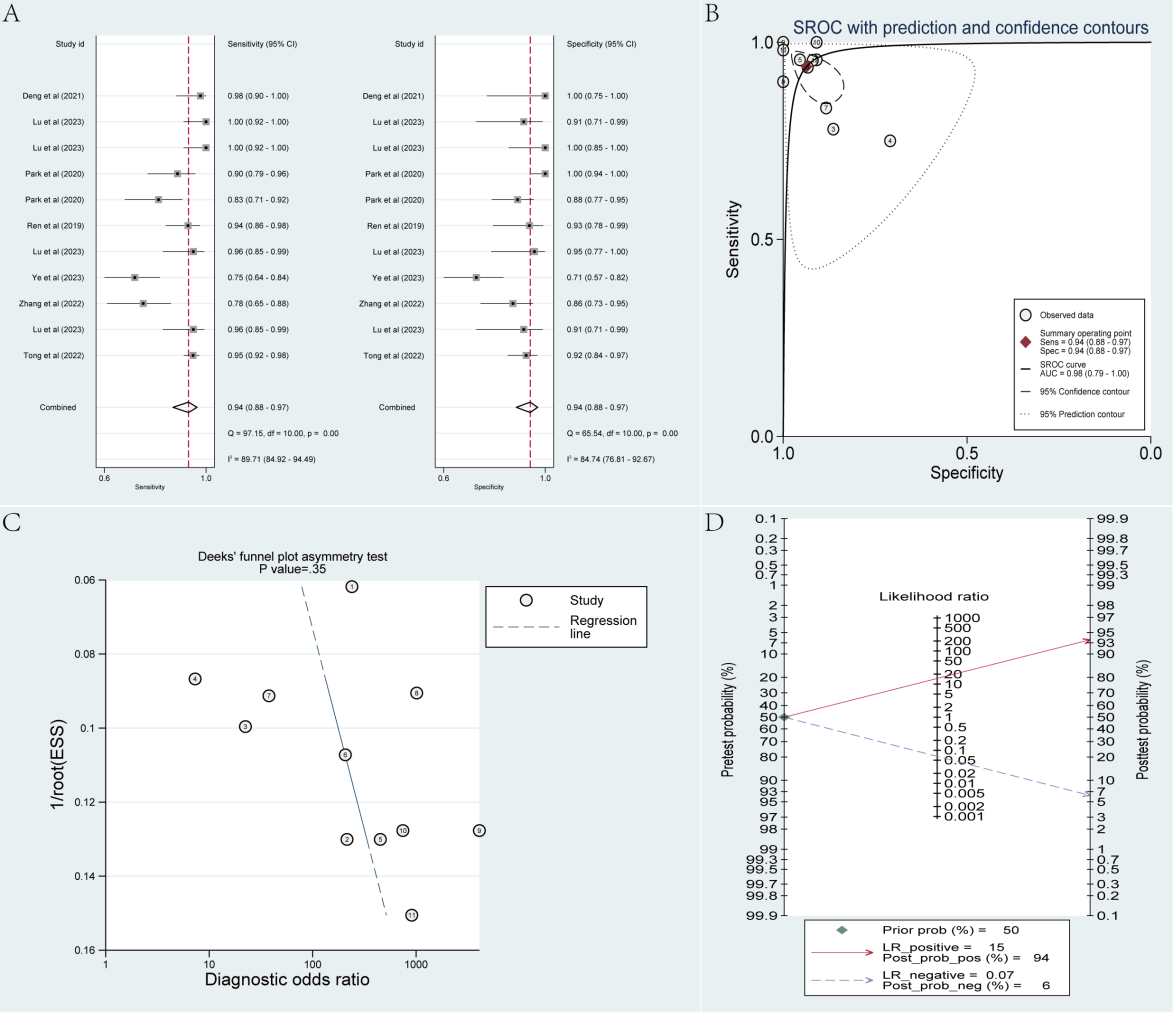
Meta-analysis results of radiomics-based machine learning models in differentiating pancreatic ductal adenocarcinoma from mass-forming pancreatitis within the training set. Data were derived from studies [25,26,28,30,32,35,40]. (**A**) Coupled forest plots of pooled sensitivity and specificity. (**B**) Summary receiver operating characteristic curve. (**C**) Deeks’ funnel plot. (**D**) Fagan nomogram. AUC: area under the curve; ESS: effective sample size; LR: logistic regression; Prob: probability; Sens: sensitivity; Spec: specificity; SROC: summary receiver operating characteristic.

The training set included 11 radiomics-based ML models. By using a bivariate mixed-effects model, the pooled sensitivity, specificity, SROC AUC, PLR, NLR, and DOR were calculated as 0.94 (95% CI 0.88‐0.97), 0.94 (95% CI 0.88‐0.97), 0.98 (95% CI 0.79‐1.00), 14.8 (95% CI 7.3‐29.8), 0.07 (95% CI 0.03‐0.13), and 225 (95% CI 63‐807), respectively. According to the funnel plot, no noticeable publication bias was observed across the included studies. In our analysis, PDAC accounted for approximately 50% (7635/14,406) of the cases. Taking 50% as the previous probability for the disease, given a PLR of 15, a positive result (PDAC) predicted by the models would correspond to a 94% probability of true PDAC. With an NLR of 0.07, a negative result (MFP) predicted by the models would imply a 94% probability of true MFP (Figure 2).

#### Independent Validation Set

**Figure 3.**
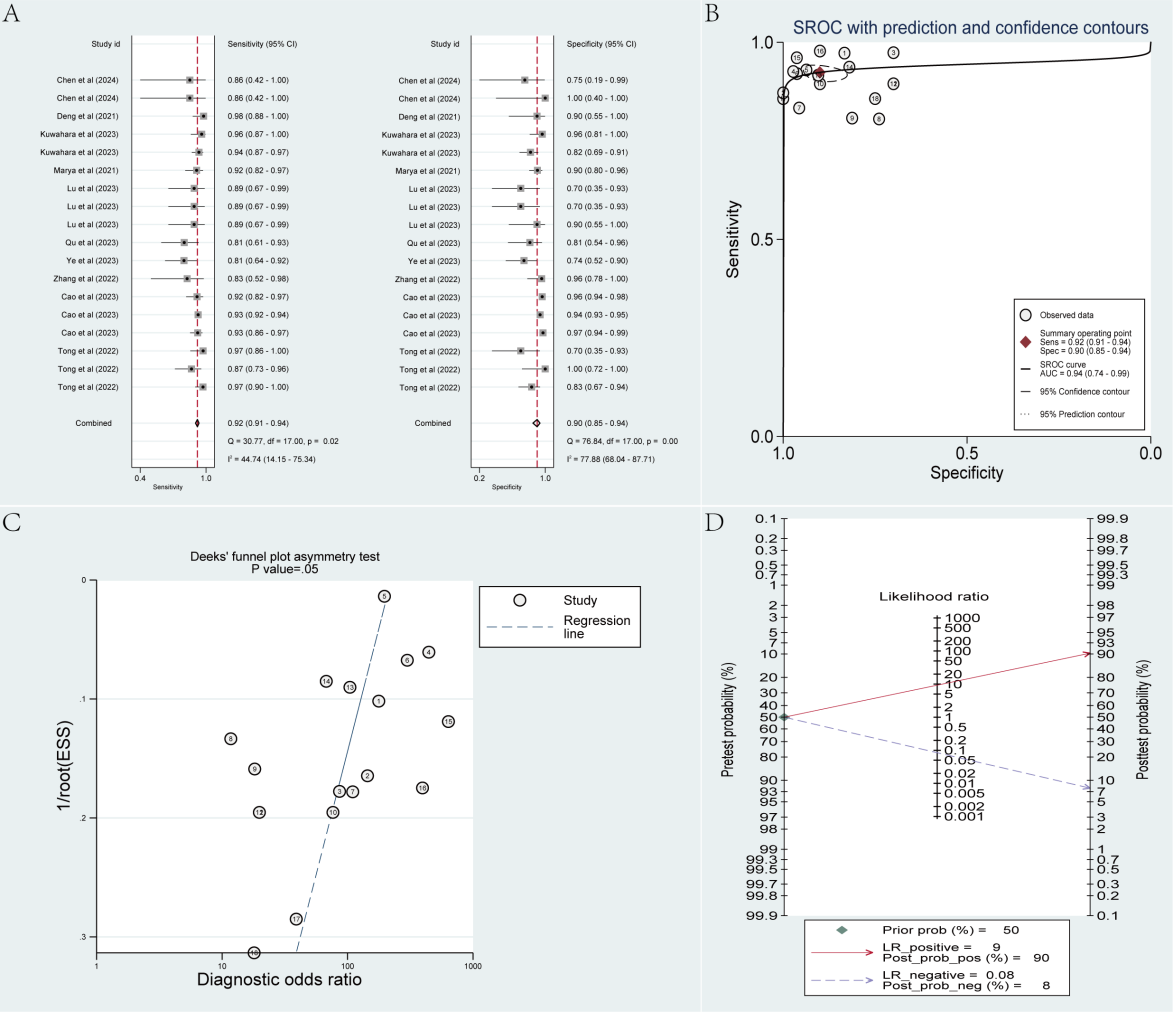
Meta-analysis results of radiomics-based machine learning models in differentiating pancreatic ductal adenocarcinoma from mass-forming pancreatitis within the independent validation set. Data were derived from studies [25,26,29,32,35,40-42,45,46]. (**A**) Coupled forest plots of pooled sensitivity and specificity. (**B**) Summary receiver operating characteristic curve. (**C**) Deeks’ funnel plot. (**D**) Fagan nomogram. AUC: area under the curve; ESS: effective sample size; LR: logistic regression; Prob: probability; Sens: sensitivity; Spec: specificity; SROC: summary receiver operating characteristic.

The independent validation set included 18 radiomics-based ML models. The pooled sensitivity, specificity, SROC AUC, PLR, NLR, and DOR, derived from a bivariate mixed-effects model, were 0.92 (95% CI 0.91‐0.94), 0.90 (95% CI 0.85‐0.94), 0.94 (95% CI 0.74‐0.99), 9.3 (95% CI 6.0‐14.2), 0.08 (95% CI 0.07‐0.11), and 110 (95% CI 62‐194), respectively. According to the funnel plot, no noticeable publication bias was noticed among the included studies. In our study, PDAC accounted for approximately 50% (7635/14,406) of the cases. Taking 50% as the previous probability for the disease, given a PLR of 9, a positive result (PDAC) predicted by the models would imply a 90% probability of true PDAC. With an NLR of 0.08, a negative result (MFP) predicted by the models would correspond to a 92% probability of true MFP (Figure 3).

#### Cross-Validation Set

**Figure 4.**
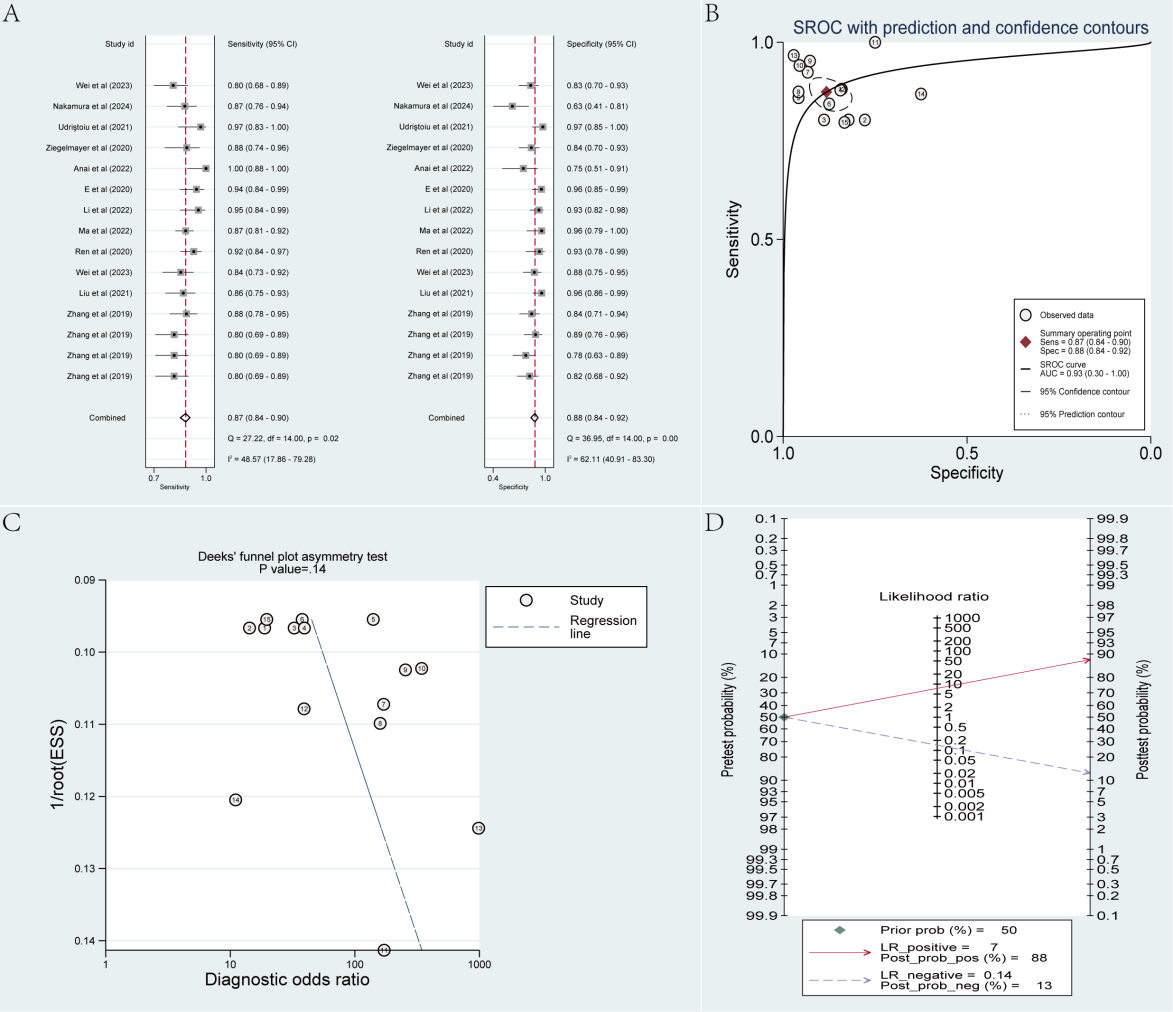
Meta-analysis results of radiomics-based machine learning models in differentiating pancreatic ductal adenocarcinoma from mass-forming pancreatitis within the cross-validation set. Data were derived from studies [23,24,31,33,34,36-39,43,44]. (**A**) Coupled forest plots of pooled sensitivity and specificity. (**B**) Summary receiver operating characteristic curve. (**C**) Deeks’ funnel plot. (**D**) Fagan nomogram. AUC: area under the curve; ESS: effective sample size; LR: logistic regression; Prob: probability; Sens: sensitivity; Spec: specificity; SROC: summary receiver operating characteristic.

The cross-validation set involved 15 radiomics-based ML models. The pooled sensitivity, specificity, SROC AUC, PLR, NLR, and DOR, calculated employing a bivariate mixed-effects model, were 0.87 (95% CI 0.84‐0.90), 0.88 (95% CI 0.84‐0.92), 0.93 (95% CI 0.30‐1.00), 7.4 (95% CI 5.2‐10.6), 0.14 (95% CI 0.11‐0.18), and 52 (95% CI 30‐90), respectively. According to the funnel plot, no evident publication bias was noted across the included studies. Approximately 50% (7635/14,406) of the cases in the analysis were PDAC. Taking 50% as the previous probability of the disease, given a PLR of 7, a positive result (PDAC) predicted by the models would have an 88% probability of true PDAC. With an NLR of 0.14, a negative result (MFP) predicted by the models would yield an 87% probability of true MFP (Figure 4).

#### Subgroup Analysis by Different Imaging Modalities

##### CT-Based ML Model

###### Training set

The training set included 8 CT-based ML models. By employing a bivariate mixed-effects model, the pooled sensitivity, specificity, SROC AUC, PLR, NLR, and DOR were calculated as 0.90 (95% CI 0.84‐0.95), 0.92 (95% CI 0.85‐0.96), 0.97 (95% CI 0.41‐1.00), 11.8 (95% CI 5.6‐24.7), 0.10 (95% CI 0.06‐0.19), and 113 (95% CI 33‐389), respectively. According to the funnel plot, no noticeable publication bias was noted across the included studies. Approximately 50% (7635/14,406) of the cases in the analysis were PDAC. Taking 50% as the previous probability for the disease, given a PLR of 12, a positive result (PDAC) predicted by the models would correspond to a 92% probability of true PDAC. With an NLR of 0.10, a negative result (MFP) predicted by the models would imply a 91% probability of true MFP (Multimedia Appendix 3).

###### Cross-validation training set

In total, 7 CT-based ML models were included in the cross-validation training set. The pooled sensitivity, specificity, SROC AUC, PLR, NLR, and DOR, computed using a bivariate mixed-effects model, were 0.91 (95% CI 0.85‐0.94), 0.89 (95% CI 0.84‐0.93), 0.95 (95% CI 0.19‐1.00), 8.6 (95% CI 5.6‐13.0), 0.11 (95% CI 0.07‐0.17), and 81 (95% CI 38‐174), respectively. Based on the funnel plot, no evident publication bias was noted among the included studies. PDAC represented about 50% of the cases in the analysis. Taking 50% as the previous probability for the disease, given a PLR of 9, a positive result (PDAC) predicted by the models would imply a 90% probability of true PDAC. With an NLR of 0.11, a negative result (MFP) predicted by the models would correspond to a 90% probability of true MFP (Multimedia Appendix 4).

###### Independent Validation set

The independent validation set involved 8 CT-based ML models. The pooled sensitivity, specificity, SROC AUC, PLR, NLR, and DOR, derived from a bivariate mixed-effects model, were 0.91 (95% CI 0.87‐0.94), 0.91 (95% CI 0.84‐0.95), 0.96 (95% CI 0.77‐0.99), 9.9 (95% CI 5.3‐18.8), 0.10 (95% CI 0.06‐0.15), and 100 (95% CI 35‐282), respectively. According to the funnel plot, no evident publication bias was noted across the included studies. In our analysis, PDAC accounted for approximately 50% (7635/14,406) of the cases. Taking 50% as the previous probability for the disease, given a PLR of 10, a positive result (PDAC) predicted by the models would correspond to a 91% probability of true PDAC. With an NLR of 0.10, a negative result (MFP) predicted by the models would correspond to a 91% probability of true MFP (Multimedia Appendix 5).

### EUS-Based ML Model

#### Independent Validation Set

A total of 6 EUS-based ML models were involved in the independent validation set. The pooled sensitivity, specificity, SROC AUC, PLR, NLR, and DOR, calculated employing a bivariate mixed-effects model, were 0.94 (95% CI 0.91‐0.96), 0.88 (95% CI 0.80‐0.93), 0.97 (95% CI 0.78‐1.00), 7.7 (95% CI 4.7‐12.5), 0.07 (95% CI 0.04‐0.11), and 114 (95% CI 60‐217), respectively. The funnel plot implied no marked publication bias across the included studies. In our analysis, PDAC accounted for approximately 50% (7635/14,406) of the cases. Taking 50% as the previous probability of the disease, given a PLR of 8, a positive result (PDAC) predicted by the models would indicate an 88% probability of true PDAC. With an NLR of 0.07, a negative result (MFP) predicted by the models would correspond to a 94% probability of true MFP (Multimedia Appendix 6).

### PET-CT–Based ML Model

#### Cross-Validation Training Set

In total, 7 PET-CT–based ML models were included in the cross-validation training set. The pooled sensitivity, specificity, SROC AUC, PLR, NLR, and DOR, derived from a bivariate mixed-effects model, were 0.84 (95% CI 0.80‐0.87), 0.88 (95% CI 0.82‐0.92), 0.88 (95% CI 0.27‐0.99), 7.1 (95% CI 4.7‐10.9), 0.19 (95% CI 0.15‐0.23), and 38 (95% CI 22‐68), respectively. The funnel plot revealed no notable publication bias across the included studies. Approximately 50% (7635/14,406) of the cases in the analysis were PDAC. Taking 50% as the previous probability for the disease, given a PLR of 7, a positive result (PDAC) predicted by the models would correspond to an 88% probability of true PDAC. With an NLR of 0.19, a negative result (MFP) predicted by the models would correspond to an 84% probability of true MFP (Multimedia Appendix 7).

### MRI-Based ML Model

According to Shiraishi et al [27], linear and nonlinear SVM models yielded AUCs of 0.89 and 0.82 in the training set, and 0.74 and 0.96 in the cross-validation set, respectively. In the testing set, the nonlinear SVM outperformed the linear SVM, with an AUC of 96.2% versus 74.4%. Deng et al [35] also employed an SVM model in their study, where the AUCs for the T1-weighted imaging, T2-weighted imaging, arterial phase, portal phase, and clinical models were 0.89, 0.91, 0.96, 0.99, and 0.52 in the training set, and 0.88, 0.90, 0.92, 0.96, and 0.65 in the independent validation set. All models surpassed radiologists and models based on clinical data in both sets. Chen et al [41] found that the accuracy and AUCs of the previous difference guidance network combined lesion and background information were 87.5% and 89.98% in conventional networks, and 89.77% and 92.8% in advanced networks.

## Discussion

### Principal Findings

This meta-analysis systematically evaluates the ability of radiomics-based ML models to distinguish PDAC from MFP. The results indicate that the pooled sensitivity, specificity, and SROC AUC in the independent validation set are 0.92, 0.90, and 0.94, respectively. These findings underscore the ability of radiomics-based ML models as a noninvasive diagnostic tool for distinguishing between PDAC and MFP, potentially decreasing the need for invasive procedures like EUS-FNA.

### Comparison With Previous Work

Several investigators have summarized the use of single imaging modalities, like CT or EUS, based on conventional imaging characteristics for differentiating PDAC from MFP. Their results indicate a promising diagnostic accuracy. For instance, Yoon et al [47] have reported that CT achieved a sensitivity of 0.83 (95% CI 0.75‐0.90) and a specificity of 0.85 (95% CI 0.81‐0.89), demonstrating its ability to detect pancreatic lesions. According to Yang et al [48], no noticeable difference in diagnostic performance is noted between EUS and CT. The sensitivity, specificity, and AUC are 0.82 (95% CI 0.73‐0.88), 0.95 (95% CI 0.90‐0.97), and 0.90 (95% CI, 0.87‐92) for EUS, respectively, and 0.81 (95% CI 0.75‐0.85), 0.94 (95% CI 0.90‐0.96), and 0.92 (95% CI 0.90‐0.94) for CT, respectively. However, these studies rely on traditional imaging features that are often subjectively interpreted. In contrast, our study applies radiomics-based approaches to the same imaging modalities, extracting high-dimensional quantitative data to provide a more objective and nuanced analysis. By incorporating multiple imaging sources and advanced diagnostic models, our study extends these findings and provides broader evidence, highlighting the superior predictive performance of radiomics-based ML models in supporting clinical decision-making to distinguish PDAC from MFP.

### Future Directions

In this study, the diagnostic imaging modalities for PDAC primarily include CT, MRI, EUS, and PET-CT. According to the 2023 European Society for Medical Oncology Clinical Practice Guideline for pancreatic cancer [49], these imaging modalities play distinct roles in diagnosis and management. CT, as the recommended first-line imaging tool, provides a robust assessment of tumor size, vascular involvement, and metastatic disease. However, it has limitations in detecting isoattenuating tumors and small metastases. MRI, with superior soft-tissue contrast, complements CT, especially in complex cases or when contrast-enhanced imaging is contraindicated. EUS offers high-resolution images, making it essential for clarifying indeterminate findings. However, its application remains challenging due to its reliance on operators and limitations in evaluating distant metastases. PET-CT, although less commonly used for initial diagnosis, offers critical metabolic insights for identifying ambiguous or metastatic lesions. Each modality has its own strengths and limitations, and thus, the use of these modalities should be tailored for different patients in clinical practice.

Our meta-analysis highlights the effectiveness of radiomics-based ML models in differentiating PDAC from MFP. The pooled sensitivity and specificity for CT-based ML models exceed 90%, establishing CT as a reliable primary diagnostic tool. The AUCs for MRI-based ML models consistently are above 0.9, confirming their role in detailed tissue characterization. EUS-based ML models exhibited the highest sensitivity (94%) and were particularly effective for detecting subtle or challenging lesions. PET-CT–based ML models, despite moderate diagnostic metrics, provide valuable information on metabolism for ambiguous cases. According to these findings, CT could be used for initial screening of tumor size, vascular involvement, and metastatic disease, MRI for enhanced evaluation of complex cases, EUS for histological confirmation in indeterminate cases, and PET-CT for identifying ambiguous or metastatic lesions. This approach maximizes diagnostic precision and integrates radiomics effectively into clinical workflows.

Beyond diagnostic performance, real-world implementation of these imaging modalities also depends on factors like accessibility, cost-effectiveness, and ease of integration into routine practice. CT is widely accessible, relatively low-cost, and fast, making it suitable as a frontline diagnostic tool in most clinical settings. MRI offers superior tissue characterization but is more expensive and less available in resource-limited environments. EUS requires specialized equipment and expertise, often limiting its use to tertiary centers, while PET-CT is costly and not routinely employed in initial diagnostic workflows. Thus, while radiomics-based ML models show promising accuracy across modalities, their clinical utility must be contextualized based on resource availability, local expertise, and patient-specific considerations. Future implementation should align model development with practical deployment strategies to optimize real-world adoption.

Radiomics alone has demonstrated considerable diagnostic performance in distinguishing PDAC from MFP. However, only a few studies use both clinical features and radiomics features to construct models. Clinical data (ie, patient age, serum markers, and medical history) can help reveal disease heterogeneity that may not be reflected in imaging features. Incorporating these features could significantly enhance model performance [21,50]. Such integration may also improve clinical interpretability and support decision-making, especially in borderline or ambiguous cases where radiomics alone might be insufficient. Nonetheless, combining multimodal data introduces challenges, like increased model complexity, the risk of overfitting with limited sample sizes, and the need for standardized clinical data collection. Future studies should focus on developing interpretable multidomain models and prospective data integration strategies that align with real-world clinical workflows, thereby enhancing both diagnostic accuracy and clinical applicability.

Multicenter and multiregional validations are crucial for enhancing the generalizability of radiomics-based ML models, as they provide a more comprehensive assessment of model performance across diverse clinical settings [21,51]. In this meta-analysis, most studies rely on single-center designs with internal validation, such as random sampling or cross-validation, which may not fully capture the variability encountered in different clinical settings. The use of single-center data can introduce bias, as it may not account for differences in imaging equipment, acquisition protocols, and patient populations that are present in real-world clinical practice.

Future studies should prioritize multicenter collaboration and the use of independent external validation sets to ensure broader representativeness. To address heterogeneity across centers, standardized imaging acquisition protocols (eg, harmonized contrast phases and MRI sequences) and consistent reporting of imaging parameters are essential. Image preprocessing methods (such as voxel resampling and intensity normalization) and harmonization techniques (eg, ComBat) can further mitigate scanner-related variability. In addition, the adoption of consensus-based guidelines for segmentation, feature extraction, and model evaluation would facilitate methodological consistency and improve reproducibility. These practical measures are essential to support the robust and clinically scalable deployment of radiomics-based ML models.

Given the relatively low incidence of PDAC, many studies included in this meta-analysis have limited sample sizes, which could contribute to selection bias and affect model robustness. The diagnostic performance of radiomics-based ML models is closely tied to sample sizes. Therefore, future multicenter studies with larger sample sizes are warranted. In addition, techniques, such as data augmentation (eg, image enhancement and synthetic data generation), can help expand datasets to improve the generalizability and stability of models in clinical practice.

In this meta-analysis, most included studies use traditional ML approaches, like SVM and RF, to construct radiomics-based ML models. While these methods can achieve high diagnostic accuracy, they often rely on manual feature extraction, which may lead to information loss [52]. This limitation can affect the generalizability of radiomics-based ML models across different clinical settings.

In contrast, DL techniques like convolutional neural networks can automatically learn features from raw images, offering a more comprehensive feature representation [52]. Although relatively few studies in our analysis use DL to construct models, the results are promising, indicating the potential advantages of DL in capturing complex imaging patterns. Future studies should explore advanced DL methods, like multimodal learning and transfer learning, to improve the diagnostic performance of radiomics-based ML models by integrating diverse imaging data [53]. This could enhance the adaptability and clinical utility of these models in differentiating PDAC from MFP. According to the RQS, the score for the included studies ranges from 5 points (14%) to 17 points (47%), with an average score of 9 (25%). This relatively low-to-moderate scoring reflects the stringency of the RQS framework. It places particular emphasis on prospective design, biological validation, repeatability assessment, and data or code availability. However, these factors are not widely adopted in radiomics research. While lower RQS scores suggest areas for improvement, they should be interpreted with caution. Most included studies fulfilled essential technical criteria, like detailed imaging protocols, appropriate feature selection methods, and validation, using independent datasets. These strengths underpin the robustness of the diagnostic accuracy estimates in this meta-analysis. However, the absence of phantom studies, limited use of longitudinal imaging, and inadequate reporting of model calibration or cost-effectiveness may limit model reproducibility and cross-center generalizability. Future radiomics studies should aim for improved adherence to reporting guidelines and quality frameworks to enhance transparency, clinical translatability, and model deployment across heterogeneous clinical settings.

### Strengths and Limitations

This study represents the first systematic appraisal of radiomics-based ML models in distinguishing PDAC from MFP across multiple imaging modalities, providing robust evidence for noninvasive diagnostic strategies. By synthesizing data from various studies, it offers critical insights into the clinical application and development of radiomics-based ML models in the future.

However, several limitations should be considered. First, despite a vast and systematic search, the number of included studies is relatively small, limiting detailed subgroup analyses of different radiomics-based ML models and reducing the depth of discussions on specific diagnostic approaches. This limitation may affect the generalizability of the findings to diverse clinical settings. Second, substantial heterogeneity remains a challenge across the included studies. Differences in imaging protocols, segmentation methods (manual vs semiautomatic), feature selection strategies (such as least absolute shrinkage and selection operator and minimum redundancy maximum relevance), classifier types (such as logistic regression, decision tree, SVM, adaptive boosting, RF, and DL), and validation strategies all contribute to methodological variability. Many pipelines also rely on investigator-defined parameters, which may introduce bias. While most studies employ internal validation methods, like random sampling or cross-validation, only a limited number have implemented independent external or multicenter validation, limiting the generalizability of model performance across diverse clinical environments. These issues highlight the urgent need for standardized reporting guidelines and harmonized study designs to ensure the reproducibility and clinical applicability of radiomics-based ML models. Future studies should prioritize multicenter collaboration and adopt standardized radiomics pipelines to reduce heterogeneity and enhance generalizability. Third, although subgroup analyses are executed by imaging modality, these are descriptive in nature and employ separate bivariate models for each modality. No formal statistical comparisons between subgroups or adjustments for multiple comparisons are performed. This approach is chosen to examine diagnostic performance trends across modalities without overinterpreting differences, given the limited number of studies per subgroup and inherent heterogeneity. Future meta-analyses with larger sample sizes may benefit from formal comparative subgroup analyses.Finally, certain imaging modalities are less representative, such as MRI and PET-CT, making it challenging to fully appraise their diagnostic performance and potentially influencing the pooled diagnostic estimates. Finally, our meta-analysis does not describe imaging parameters across studies in detail, such as the specific phases used in contrast-enhanced CT (like arterial and venous) and the types of MRI sequences used (like T1-weighted and T2-weighted). This may hinder the assessment of consistency and reproducibility across studies.

### Conclusions

Radiomics-based ML models demonstrate excellent diagnostic accuracy in differentiating PDAC from MFP, supporting their potential role as noninvasive tools in clinical practice. However, to facilitate real-world adoption, future research should prioritize multicenter validation, standardized imaging protocols, and reproducible model pipelines.

## Supplementary material

10.2196/72420 Multimedia Appendix 1 Search strategy.

10.2196/72420 Multimedia Appendix 2 Radiomics quality score of the included studies.

10.2196/72420 Multimedia Appendix 3 Meta-analysis results of radiomics-based machine learning models in differentiating pancreatic ductal adenocarcinoma from mass-forming pancreatitis within the computed tomography training set. (A) Coupled forest plots of pooled sensitivity and specificity. (B) Summary receiver operating characteristic curve. (C) Deeks’ funnel plot. (D) Fagan nomogram.

10.2196/72420 Multimedia Appendix 4 Meta-analysis results of radiomics-based machine learning models in differentiating pancreatic ductal adenocarcinoma from mass-forming pancreatitis within the computed tomography cross-validation set. (A) Coupled forest plots of pooled sensitivity and specificity. (B) Summary receiver operating characteristic curve. (C) Deeks’ funnel plot. (D) Fagan nomogram.

10.2196/72420 Multimedia Appendix 5 Meta-analysis results of radiomics-based machine learning models in differentiating pancreatic ductal adenocarcinoma from mass-forming pancreatitis within the computed tomography independent validation set. (A) Coupled forest plots of pooled sensitivity and specificity. (B) Summary receiver operating characteristic curve. (C) Deeks’ funnel plot. (D) Fagan nomogram.

10.2196/72420 Multimedia Appendix 6 Meta-analysis results of radiomics-based machine learning models in differentiating pancreatic ductal adenocarcinoma from mass-forming pancreatitis within the endoscopic ultrasound independent validation set. (A) Coupled forest plots of pooled sensitivity and specificity. (B) Summary receiver operating characteristic curve. (C) Deeks’ funnel plot. (D) Fagan nomogram.

10.2196/72420 Multimedia Appendix 7 Meta-analysis results of radiomics-based machine learning models in differentiating pancreatic ductal adenocarcinoma from mass-forming pancreatitis within the positron emission tomography-computed tomography cross-validation set. (A) Coupled forest plots of pooled sensitivity and specificity. (B) Summary receiver operating characteristic curve. (C) Deeks’ funnel plot. (D) Fagan nomogram.

10.2196/72420 Checklist 1 PRISMA (Preferred Reporting Items for Systematic reviews and Meta-Analyses) Checklist.

## Abbreviations

AUC: area under the curve
CT: computed tomography
DL: deep learning
DOR: diagnostic odds ratio
EUS: endoscopic ultrasound
EUS-FNA: endoscopic ultrasound-guided fine-needle aspiration
MFP: mass-forming pancreatitis
ML: machine learning
MRI: magnetic resonance imaging
NLR: negative likelihood ratio
PDAC: pancreatic ductal adenocarcinoma
PET-CT: positron emission tomography-computed tomography
PLR: positive likelihood ratio
PRISMA: Preferred Reporting Items for Systematic reviews and Meta-Analyses
RF: random forest
RQS: radiomics quality score
SROC: summary receiver operating characteristic
SROC AUC: area under the curve of summary receiver operating characteristic
SVM: support vector machine
