# Supplementary material for: Effectiveness of Radiomics-Based Machine Learning Models in Differentiating Pancreatitis and Pancreatic Ductal Adenocarcinoma: Systematic Review and Meta-Analysis
Source: J Med Internet Res. 2025 Jul 31;27:e72420. doi: 10.2196/72420 (PMC12313348; doi:10.2196/72420)
Supplement: Multimedia Appendix 1 [file jmir-v27-e72420-s001.docx]

**Table S1** Search Strategy

Database of Systematic Reviews from the creation of the database up to June 2024.

1. **Pubmed**

| **#** | **Searches** | **Results** |
| --- | --- | --- |
| 1 | Carcinoma, Pancreatic Ductal[MeSH Terms] | 13565 |
| 2 | "duct carcinoma of the pancreas"[Title/Abstract] OR "Duct Cell Carcinoma of the Pancreas"[Title/Abstract] OR "ductal adenocarcinoma of the pancreas"[Title/Abstract] OR "ductal carcinoma of the pancreas"[Title/Abstract] OR "pancreas duct adenocarcinoma"[Title/Abstract] OR "pancreas duct carcinoma"[Title/Abstract] OR "Pancreas Duct Cell Carcinoma*"[Title/Abstract] OR "pancreas ductal adenocarcinoma"[Title/Abstract] OR "pancreas ductal carcinoma"[Title/Abstract] OR "pancreatic duct adenocarcinoma"[Title/Abstract] OR "pancreatic duct carcinogenesis"[Title/Abstract] OR "pancreatic duct carcinoma"[Title/Abstract] OR "pancreatic duct cell adenocarcinoma"[Title/Abstract] OR "pancreatic duct cell carcinoma"[Title/Abstract] OR "pancreatic ductal adenocarcinoma"[Title/Abstract] OR "pancreatic ductal cancer*"[Title/Abstract] OR "pancreatic ductal carcinoma*"[Title/Abstract] OR "PDAC"[Title/Abstract] | 17828 |
| 3 | Machine Learning[MeSH Terms] | 70548 |
| 4 | "Adaboost"[Title/Abstract] OR "AlexNet"[Title/Abstract] OR "artificial intelligence"[Title/Abstract] OR "Bayesian network"[Title/Abstract] OR "CNN"[Title/Abstract] OR "Decision tree"[Title/Abstract] OR "Deep learning"[Title/Abstract] OR "Ensemble Learning"[Title/Abstract] OR "GoogLeNet"[Title/Abstract] OR "Gradient Boosting Machine"[Title/Abstract] OR "K-Nearest Neighbor"[Title/Abstract] OR "learning machine*"[Title/Abstract] OR "LightGBM"[Title/Abstract] OR "machine learning"[Title/Abstract] OR "Multilayer perceptron"[Title/Abstract] OR "Naive Bayesian"[Title/Abstract] OR "neural network*"[Title/Abstract] OR "Nomogram"[Title/Abstract] OR "Radiomic*"[Title/Abstract] OR "random forest"[Title/Abstract] OR "ResNet"[Title/Abstract] OR "Support vector machine"[Title/Abstract] OR "SVM"[Title/Abstract] OR "Texture"[Title/Abstract] OR "Transfer Learning"[Title/Abstract] OR "VGGNet"[Title/Abstract] OR "XGBoost"[Title/Abstract] | 375309 |
| 5 | (#1 OR #2) AND (#3 OR #4) | 680 |

1. **Embase**

| **#** | **Searches** | **Results** |
| --- | --- | --- |
| 1 | 'pancreatic ductal carcinoma'/exp | 10074 |
| 2 | 'duct carcinoma of the pancreas':ab,ti OR 'duct cell carcinoma of the pancreas':ab,ti OR 'ductal adenocarcinoma of the pancreas':ab,ti OR 'ductal carcinoma of the pancreas':ab,ti OR 'pancreas duct adenocarcinoma':ab,ti OR 'pancreas duct carcinoma':ab,ti OR 'pancreas duct cell carcinoma*':ab,ti OR 'pancreas ductal adenocarcinoma':ab,ti OR 'pancreas ductal carcinoma':ab,ti OR 'pancreatic duct adenocarcinoma':ab,ti OR 'pancreatic duct carcinogenesis':ab,ti OR 'pancreatic duct carcinoma':ab,ti OR 'pancreatic duct cell adenocarcinoma':ab,ti OR 'pancreatic duct cell carcinoma':ab,ti OR 'pancreatic ductal adenocarcinoma':ab,ti OR 'pancreatic ductal cancer*':ab,ti OR 'pancreatic ductal carcinoma*':ab,ti OR 'pdac':ab,ti | 30566 |
| 3 | 'machine learning'/exp | 487608 |
| 4 | 'adaboost':ab,ti OR 'alexnet':ab,ti OR 'artificial intelligence':ab,ti OR 'bayesian network':ab,ti OR 'cnn':ab,ti OR 'decision tree':ab,ti OR 'deep learning':ab,ti OR 'ensemble learning':ab,ti OR 'googlenet':ab,ti OR 'gradient boosting machine':ab,ti OR 'k-nearest neighbor':ab,ti OR 'learning machine*':ab,ti OR 'lightgbm':ab,ti OR 'machine learning':ab,ti OR 'multilayer perceptron':ab,ti OR 'naive bayesian':ab,ti OR 'neural network*':ab,ti OR 'nomogram':ab,ti OR 'radiomic*':ab,ti OR 'random forest':ab,ti OR 'resnet':ab,ti OR 'support vector machine':ab,ti OR 'svm':ab,ti OR 'texture':ab,ti OR 'transfer learning':ab,ti OR 'vggnet':ab,ti OR 'xgboost':ab,ti | 369690 |
| 5 | (#1 OR #2) AND (#3 OR #4) | 1281 |

1. **Cochrane**

| **#** | **Searches** | **Results** |
| --- | --- | --- |
| 1 | MeSH descriptor: [Carcinoma, Pancreatic Ductal] explode all trees | 226 |
| 2 | ('duct carcinoma of the pancreas' OR 'Duct Cell Carcinoma of the Pancreas' OR 'ductal adenocarcinoma of the pancreas' OR 'ductal carcinoma of the pancreas' OR 'pancreas duct adenocarcinoma' OR 'pancreas duct carcinoma' OR 'Pancreas Duct Cell Carcinoma*' OR 'pancreas ductal adenocarcinoma' OR 'pancreas ductal carcinoma' OR 'pancreatic duct adenocarcinoma' OR 'pancreatic duct carcinogenesis' OR 'pancreatic duct carcinoma' OR 'pancreatic duct cell adenocarcinoma' OR 'pancreatic duct cell carcinoma' OR 'pancreatic ductal adenocarcinoma' OR 'pancreatic ductal cancer*' OR 'pancreatic ductal carcinoma*' OR 'PDAC'):ab,ti | 1157 |
| 3 | MeSH descriptor: [Machine Learning] explode all trees | 986 |
| 4 | ('Adaboost' OR 'AlexNet' OR 'artificial intelligence' OR 'Bayesian network' OR 'CNN' OR 'Decision tree' OR 'Deep learning' OR 'Ensemble Learning' OR 'GoogLeNet' OR 'Gradient Boosting Machine' OR 'K-Nearest Neighbor' OR 'learning machine*' OR 'LightGBM' OR 'machine learning' OR 'Multilayer perceptron' OR 'Naive Bayesian' OR 'neural network*' OR 'Nomogram' OR 'Radiomic*' OR 'random forest' OR 'ResNet' OR 'Support vector machine' OR 'SVM' OR 'Texture' OR 'Transfer Learning' OR 'VGGNet' OR 'XGBoost'):ab,ti | 14584 |
| 5 | (#1 OR #2) AND (#3 OR #4) | 40 |

1. **Web of Science**

| **#** | **Searches** | **Results** |
| --- | --- | --- |
| 1 | duct carcinoma of the pancreas (Topic) OR Duct Cell Carcinoma of the Pancreas (Topic) OR ductal adenocarcinoma of the pancreas (Topic) OR ductal carcinoma of the pancreas (Topic) OR pancreas duct adenocarcinoma (Topic) OR pancreas duct carcinoma (Topic) OR Pancreas Duct Cell Carcinoma* (Topic) OR pancreas ductal adenocarcinoma (Topic) OR pancreas ductal carcinoma (Topic) OR pancreatic duct adenocarcinoma (Topic) OR pancreatic duct carcinogenesis (Topic) OR pancreatic duct carcinoma (Topic) OR pancreatic duct cell adenocarcinoma (Topic) OR pancreatic duct cell carcinoma (Topic) OR pancreatic ductal adenocarcinoma (Topic) OR pancreatic ductal cancer* (Topic) OR pancreatic ductal carcinoma* (Topic) OR PDAC (Topic) | 28345 |
| 2 | Adaboost (Topic) OR AlexNet (Topic) OR artificial intelligence (Topic) OR Bayesian network (Topic) OR CNN (Topic) OR Decision tree (Topic) OR Deep learning (Topic) OR Ensemble Learning (Topic) OR GoogLeNet (Topic) OR Gradient Boosting Machine (Topic) OR K-Nearest Neighbor (Topic) OR learning machine* (Topic) OR LightGBM (Topic) OR machine learning (Topic) OR Multilayer perceptron (Topic) OR Naive Bayesian (Topic) OR neural network* (Topic) OR Nomogram (Topic) OR Radiomic* (Topic) OR random forest (Topic) OR ResNet (Topic) OR Support vector machine (Topic) OR SVM (Topic) OR Texture (Topic) OR Transfer Learning (Topic) OR VGGNet (Topic) OR XGBoost (Topic) | 1663711 |
| 3 | #2 AND #1 | 898 |
