# Supplementary material for: Effectiveness of Radiomics-Based Machine Learning Models in Differentiating Pancreatitis and Pancreatic Ductal Adenocarcinoma: Systematic Review and Meta-Analysis
Source: J Med Internet Res. 2025 Jul 31;27:e72420. doi: 10.2196/72420 (PMC12313348; doi:10.2196/72420)
Supplement: Multimedia Appendix 2 [file jmir-v27-e72420-s002.docx]

**Table S2** Radiomics Quality Score (RQS) of Included Studies

| No | First Author | Year | v1 | v2 | v3 | v4 | v5 | v6 | v7 | v8 | v9 | v10 | v11 | v12 | v13 | v14 | v15 | v16 | Total | Proportion |
| --- | --- | --- | --- | --- | --- | --- | --- | --- | --- | --- | --- | --- | --- | --- | --- | --- | --- | --- | --- | --- |
| 1 | **Shuai Ren** | 2020 | 2 | 0 | 0 | 0 | 0 | 0 | 0 | 0 | 2 | 0 | 0 | 2 | 0 | 0 | 0 | 0 | 6 | 16.67 |
| 2 | **Yuquan Zhang** | 2019 | 2 | 1 | 0 | 0 | 0 | 0 | 0 | 0 | 2 | 0 | 0 | 2 | 0 | 0 | 0 | 0 | 7 | 19.44 |
| 3 | **Hao Zhang** | 2022 | 2 | 1 | 0 | 0 | 0 | 0 | 0 | 1 | 2 | 0 | 0 | 4 | 0 | 2 | 0 | 0 | 12 | 33.33 |
| 4 | **Yingjian Ye** | 2023 | 2 | 1 | 0 | 0 | 0 | 1 | 0 | 1 | 2 | 2 | 0 | 4 | 2 | 2 | 0 | 0 | 17 | 47.22 |
| 5 | **MEGUMI SHIRAISHI** | 2022 | 2 | 1 | 0 | 0 | 0 | 0 | 0 | 0 | 2 | 0 | 0 | 2 | 0 | 0 | 0 | 0 | 7 | 19.44 |
| 6 | **Shuai Ren** | 2019 | 2 | 1 | 0 | 1 | 0 | 0 | 0 | 0 | 2 | 0 | 0 | 2 | 0 | 0 | 0 | 0 | 8 | 22.22 |
| 7 | **Weinuo Qu** | 2023 | 2 | 1 | 0 | 0 | 0 | 1 | 0 | 0 | 2 | 0 | 0 | 2 | 2 | 2 | 0 | 0 | 12 | 33.33 |
| 8 | **S. Park** | 2020 | 2 | 1 | 0 | 1 | 0 | 0 | 0 | 0 | 2 | 0 | 0 | 2 | 0 | 0 | 0 | 0 | 8 | 22.22 |
| 9 | **Xi Ma** | 2022 | 2 | 1 | 0 | 1 | 0 | 1 | 0 | 0 | 2 | 2 | 0 | 2 | 2 | 2 | 0 | 0 | 15 | 41.67 |
| 10 | **Jia Lu** | 2023 | 2 | 1 | 0 | 0 | 0 | 0 | 0 | 0 | 2 | 2 | 0 | 2 | 0 | 2 | 0 | 0 | 11 | 30.56 |
| 11 | **Zhaobang Liu** | 2021 | 2 | 1 | 0 | 0 | 0 | 1 | 0 | 0 | 2 | 0 | 0 | 2 | 2 | 0 | 0 | 0 | 10 | 27.78 |
| 12 | **Jing Li** | 2021 | 2 | 1 | 0 | 0 | 0 | 0 | 0 | 1 | 2 | 0 | 0 | 2 | 0 | 0 | 0 | 0 | 8 | 22.22 |
| 13 | **Yan Deng** | 2021 | 2 | 1 | 0 | 1 | 0 | 1 | 0 | 0 | 2 | 0 | 0 | 4 | 2 | 0 | 0 | 0 | 13 | 36.11 |
| 14 | **Linning E** | 2020 | 2 | 1 | 0 | 1 | 0 | 0 | 0 | 0 | 2 | 0 | 0 | 2 | 0 | 0 | 0 | 0 | 8 | 22.22 |
| 15 | **Kenta Anai** | 2022 | 2 | 1 | 0 | 0 | 0 | 0 | 0 | 0 | 2 | 0 | 0 | 2 | 0 | 0 | 0 | 0 | 7 | 19.44 |
| 16 | **Sebastian Ziegelmayer** | 2020 | 0 | 1 | 0 | 0 | 0 | 0 | 0 | 0 | 2 | 0 | 0 | 2 | 0 | 0 | 0 | 0 | 5 | 13.89 |
| 17 | **Wenting Wei** | 2023 | 2 | 1 | 0 | 0 | 0 | 0 | 0 | 0 | 2 | 0 | 0 | 2 | 0 | 0 | 0 | 0 | 7 | 19.44 |
| 18 | **Tong Tong** | 2022 | 2 | 1 | 0 | 0 | 0 | 0 | 0 | 0 | 2 | 0 | 0 | 5 | 0 | 0 | 0 | 0 | 10 | 27.78 |
| 19 | **Luda Chen** | 2024 | 0 | 1 | 0 | 0 | 0 | 0 | 0 | 0 | 2 | 0 | 0 | 2 | 0 | 0 | 0 | 0 | 5 | 13.89 |
| 20 | **Kai Cao** | 2023 | 0 | 1 | 0 | 0 | 0 | 0 | 0 | 0 | 2 | 0 | 0 | 5 | 0 | 0 | 0 | 0 | 8 | 22.22 |
| 21 | **Anca Loredana Udriştoiu** | 2021 | 2 | 1 | 0 | 0 | 0 | 0 | 0 | 0 | 2 | 0 | 0 | 2 | 0 | 0 | 0 | 0 | 7 | 19.44 |
| 22 | **Hitomi Nakamura** | 2023 | 2 | 1 | 0 | 0 | 0 | 1 | 0 | 0 | 2 | 0 | 0 | 2 | 2 | 0 | 0 | 0 | 10 | 27.78 |
| 23 | **Neil B Marya** | 2020 | 0 | 1 | 0 | 0 | 0 | 0 | 0 | 0 | 2 | 0 | 0 | 2 | 0 | 0 | 0 | 0 | 5 | 13.89 |
| 24 | **Takamichi Kuwahara** | 2022 | 2 | 1 | 0 | 0 | 0 | 0 | 0 | 0 | 2 | 0 | 0 | 2 | 0 | 0 | 0 | 0 | 7 | 19.44 |

| Criteria | | Points |
| --- | --- | --- |
| 1 | Image protocol quality -well-documented image protocols (for example, contrast, slice thickness, energy, etc.) and/or usage of public image protocols allow reproducibility/replicability | + 1 (if protocols are well-documented)  + 1 (if public protocol is used) |
| 2 | Multiple segmentations- possible actions are: segmentation by different physicians/algorithms/software, perturbing segmentations by (random) noise, segmentation at different breathing cycles. Analyse feature robustness to segmentation variabilities | +1 |
| 3 | Phantom study on all scanners-detect inter-scanner differences and vendor-dependent features. Analyse feature robustness to these sources of variability | +1 |
| 4 | Imaging at multiple time points-collect images of individuals at additional time points. Analyse feature robustness to temporal variabilities (for example, organ movement, organ expansion/shrinkage) | +1 |
| 5 | Feature reduction or adjustment for multiple testing-decreases the risk of overfitting. Overfitting is inevitable if the number of features exceeds the number of samples. Consider feature robustness when selecting features | - 3 (if neither measure is implemented)  + 3 (if either measure is implemented) |
| 6 | Multivariable analysis with non radiomics features (for example,EGFR mutation) - is expected to provide a more holistic model. Permits correlating/inferencing between radiomics and non radiomics features | +1 |
| 7 | Detect and discuss biological correlates-demonstration of phenotypic differences (possibly associated with underlying gene–protein expression patterns) deepens understanding of radiomics and biology | +1 |
| 8 | Cut-off analyses-determine risk groups by either the median, a previously published cut-offor report a continuous risk variable. Reduces the risk of reporting overly optimistic results | +1 |
| 9 | Discrimination statistics- report discrimination statistics (for example, C-statistic, ROC curve,AUC) and their statistical significance (for example,p-values, confidence intervals). One can also apply resampling method (for example, bootstrapping, cross-validation) | + 1 (if a discrimination statistic and its statistical significance are reported)  + 1 (if aresampling method technique is also applied) |
| 10 | Calibration statistics- report calibration statistics (for example, Calibration-in-the-large/slope, calibration plots) and their statistical significance (for example,P-values, confidence intervals). One  can also apply resampling method (for example, bootstrapping, cross-validation) | + 1 (if a calibration statistic and its statistical significance are reported)  + 1 (if aresampling method technique is also applied) |
| 11 | Prospective study registered in atrial database- provides the highest level of evidence supporting the clinical validity and usefulness of the radiomics biomarker | + 7 (for prospective validation of a radiomics signature in an appropriate trial) |
| 12 | Validation-the validation is performed without retraining and without adaptation of the cut-off value, provides crucial information with regard to credible clinical performance | - 5 (if validation is missing)  +2 (if validation is based on a dataset from the same institute)  + 3 (if validation is based on a dataset from another institute)  +4 (if validation is based on two datasets from two distinct institutes)  +4 (if the study validates a previously published signature)  + 5 (if validation is based on three or more datasets from distinct institutes)  *Datasets should be of comparable size and should have at least 10 events per model feature |
| 13 | Comparison to ‘gold standard’ -assess the extent to which the model agrees with/is superior to the current ‘gold standard’ method (for example,TNM-staging for survival prediction). This comparison shows the added value of radiomics | +2 |
| 14 | Potential clinical utility- report on the current and potential application of the model in a clinical setting (for example, decision curve analysis). | +2 |
| 15 | Cost-effectiveness analysis- report on the cost-effectiveness of the clinical application (for example, QALYs generated) | +1 |
| 16 | Open science and data- make code and data publicly available. Open science facilitates knowledge transfer and reproducibility of the study | + 1 (if scans are open source)  + 1 (if region of interest segmentations are open source)  + 1 (if code is open source)  + 1 (if radiomics features are calculated on a set of representative ROIs and the calculated features and representative ROIs are open source) |
| Total points (36 = 100%) | | |
