# Supplementary figures and images for: Effectiveness of Radiomics-Based Machine Learning Models in Differentiating Pancreatitis and Pancreatic Ductal Adenocarcinoma: Systematic Review and Meta-Analysis
Source: J Med Internet Res. 2025 Jul 31;27:e72420. doi: 10.2196/72420 (PMC12313348; doi:10.2196/72420)

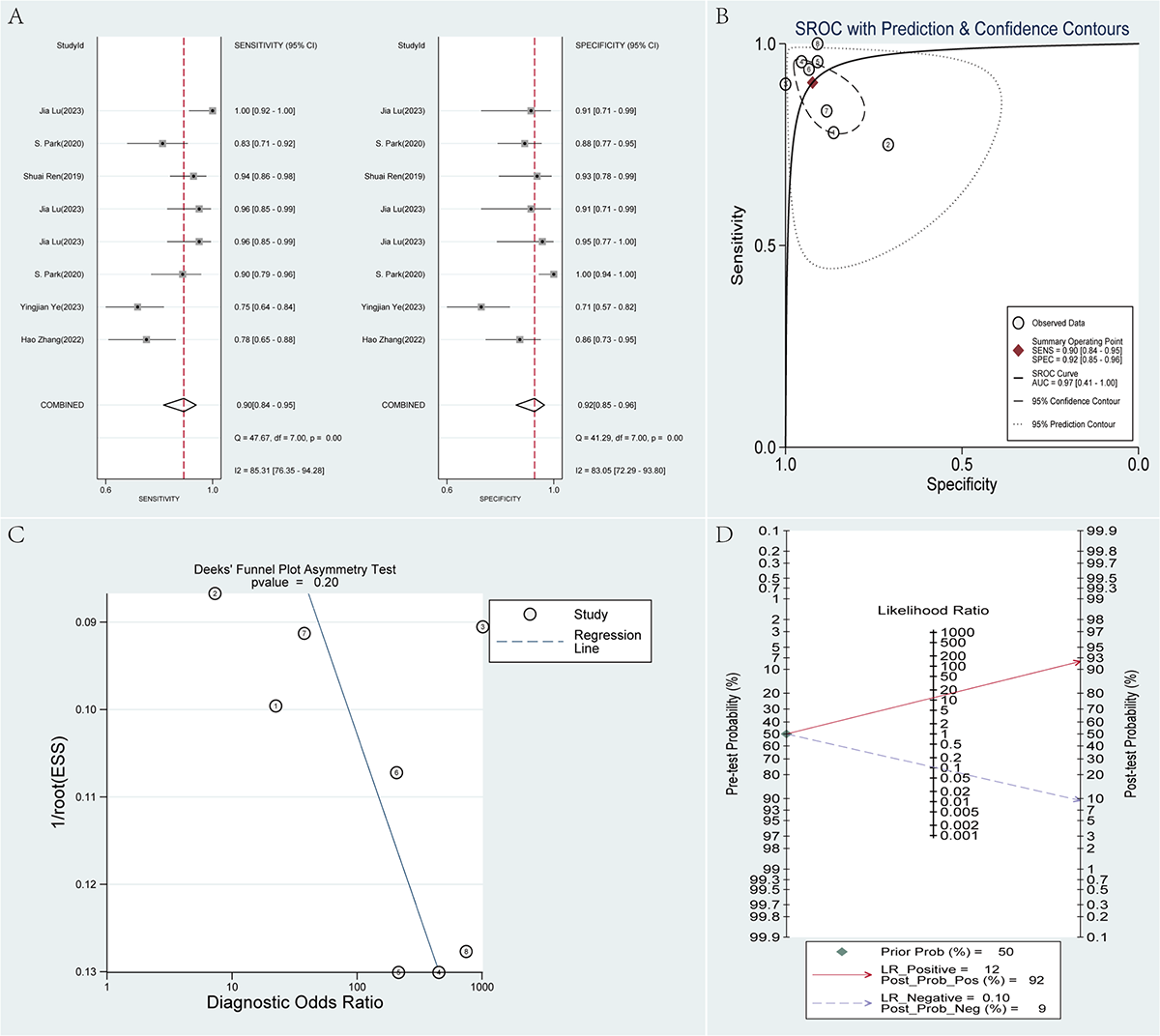

Supplement: Multimedia Appendix 3 [file jmir-v27-e72420-s003.png]

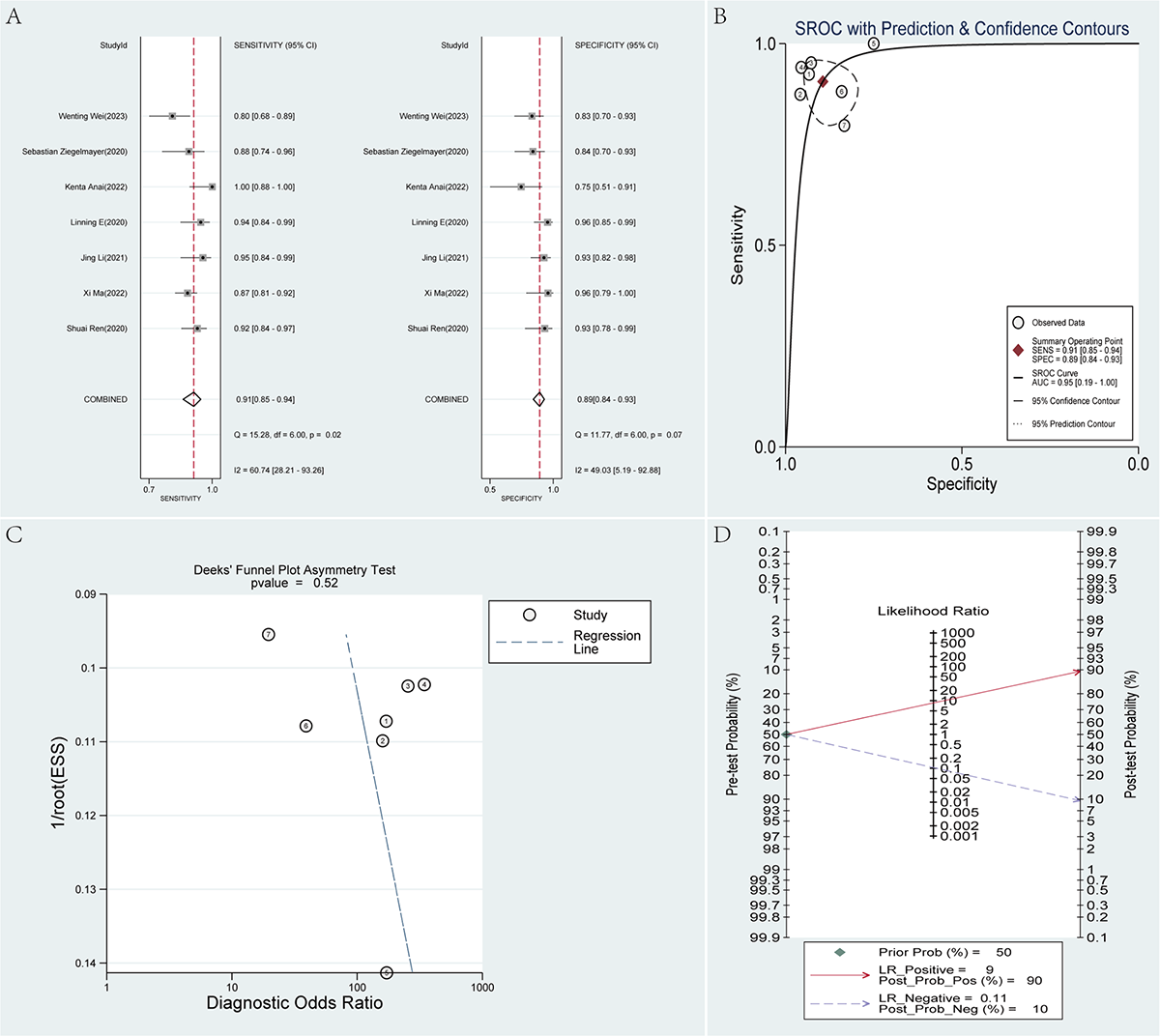

Supplement: Multimedia Appendix 4 [file jmir-v27-e72420-s004.png]

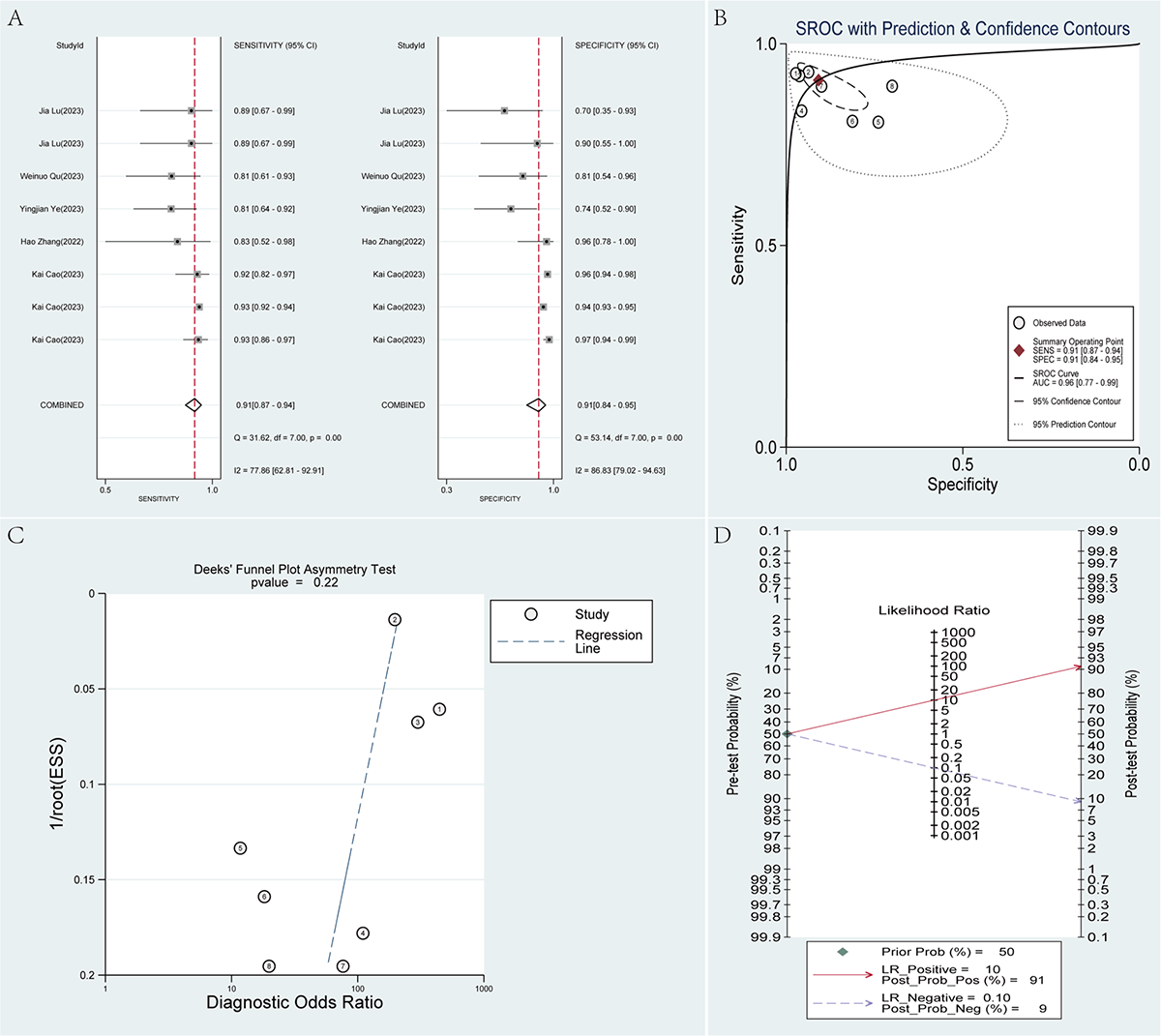

Supplement: Multimedia Appendix 5 [file jmir-v27-e72420-s005.png]

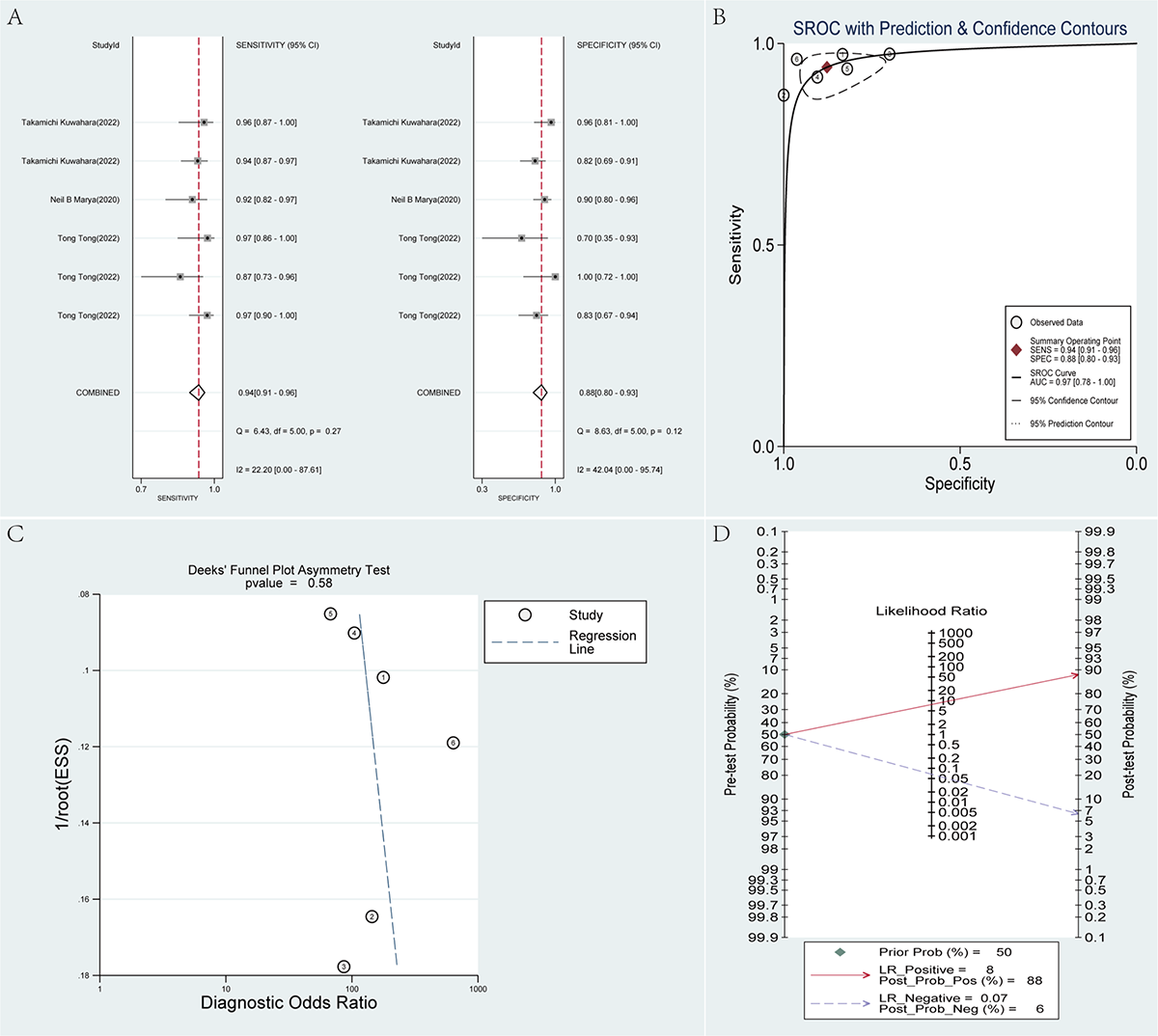

Supplement: Multimedia Appendix 6 [file jmir-v27-e72420-s006.png]

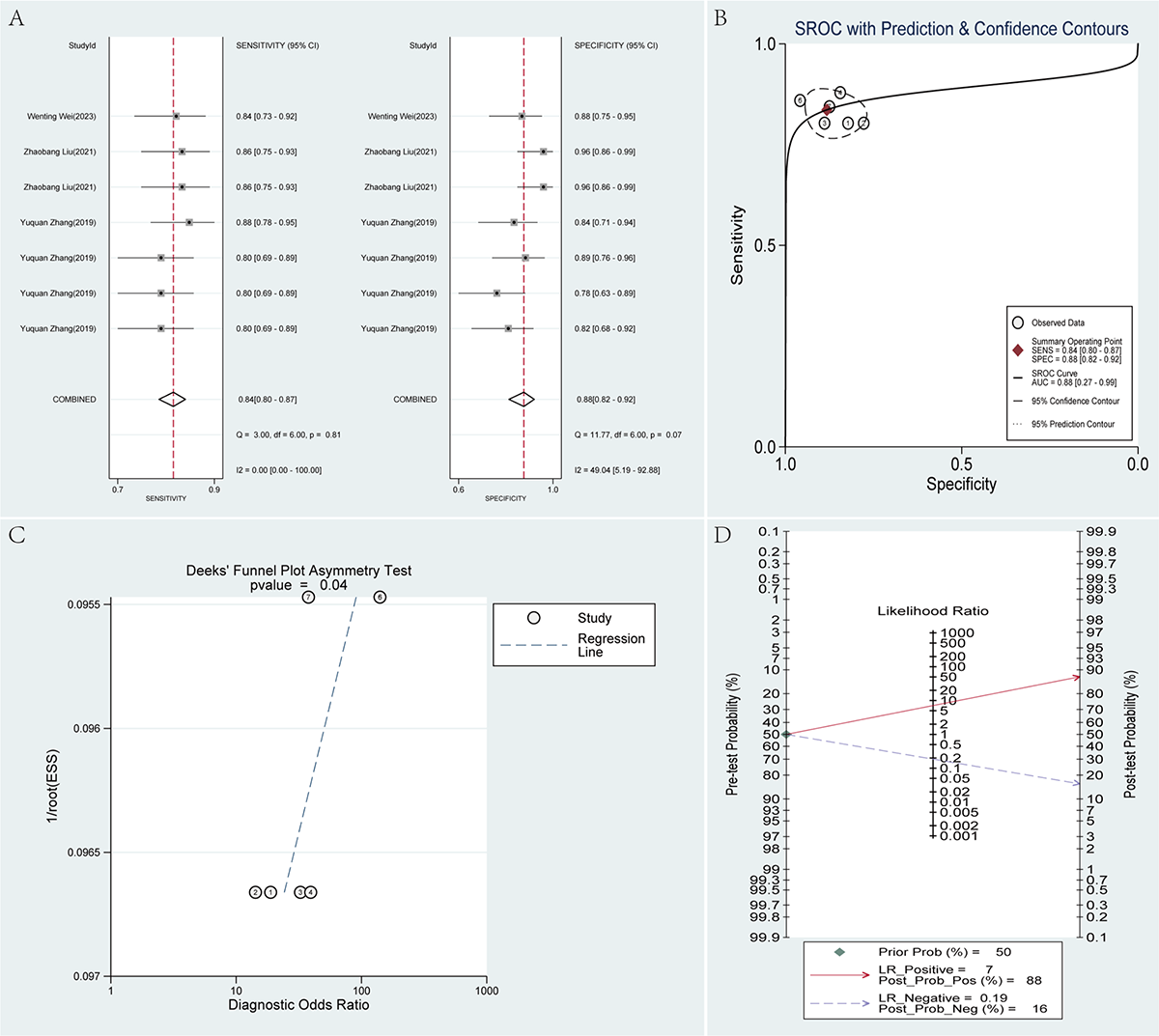

Supplement: Multimedia Appendix 7 [file jmir-v27-e72420-s007.png]
